# Data-Independent Acquisition for Improved Compound Annotation in MALDI MS Imaging

**DOI:** 10.1021/jasms.6c00128

**Published:** 2026-07-10

**Authors:** Carmen Paschke, Kerstin Strupat, Carolin M. Morawietz, Karl-Christian Schaefer, Christoph Henrich, Bernhard Spengler

**Affiliations:** 1 443714 Thermo Fisher Scientific (Bremen) GmbH, Bremen 28199, Germany; 2 Justus Liebig University, Giessen 35392, Germany; 3 TransMIT GmbH, Giessen 35392, Germany

## Abstract

Matrix-assisted laser desorption/ionization mass spectrometry imaging (MALDI MSI) enables spatial mapping of biomolecules within biological tissues, where conventional MS^1^-based workflows often result in ambiguous compound annotations. Data-dependent acquisition (DDA) can improve annotation specificity but is biased toward high-abundant ions and lacks reproducibility. Here, we present a novel MALDI MSI workflow integrating data-independent acquisition (DIA) to obtain both spatial and fragmentation information. In this approach, MS^1^ and DIA MS^2^ spectra are acquired alternately across the sample without prior knowledge of compound localization. Each image pixel consists of one MS^1^ and several DIA MS^2^ subpixels, providing both high spatial resolution for MS^1^ data and broad *m*/*z* coverage. Using small, randomized *m*/*z* isolation windows reduces spectral overlap and improves fragment-ion specificity. Data were processed using an extended Compound Discoverer, integrating mzCloud and LipidSearch for compound annotation. Applied to tissue of the parasitic worm *Fasciola hepatica*, this workflow produced detailed lipid maps and improved annotation confidence by combining precursor-mass, DIA MS^2^, and spatial-correlation information. Our results demonstrate that DIA offers a flexible strategy to integrate fragmentation information into MALDI MSI, expanding its capabilities for spatial metabolomics.

## Graphical abstract

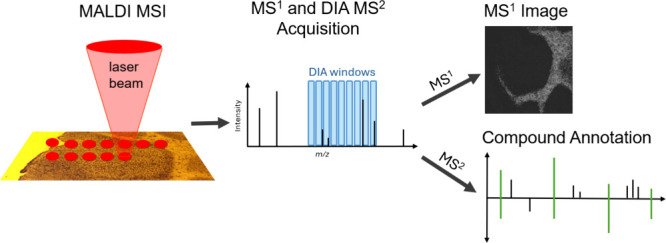

## Introduction

Investigating biological samples such as bacterial biofilms, organs (e.g., mouse brain), or whole organisms (e.g., worms) using mass spectrometry combined with spatial analysis has attracted increasing attention over the past three decades.^1–4^ Mass spectrometry Iiaging (MSI) is a powerful analytical technique that combines the high mass resolution of mass spectrometers with spatially resolved analysis.^2,3^ This enables the detection and localization of compounds within tissue samples at cellular or even subcellular level.^4,5^ Understanding the spatial distributions of metabolites and biomolecules provides important insights into biological processes and disease mechanisms, a field known as spatial metabolomics.

Several analytical ionization techniques have been adapted for MSI applications, including secondary ion mass spectrometry (SIMS), desorption electrospray ionization (DESI), and matrix-assisted laser desorption/ionization (MALDI).^4^ In this study, MALDI was used as the ionization method for MSI experiments. The MALDI technique is an ionization method of choice for MSI because it enables detection of intact biomolecules such as carbohydrates, lipids, peptides, and proteins with minimal fragmentation.^6,7^ Its sample preparation is relatively simple: the sample is cocrystallized with a matrix compound that strongly absorbs short laser pulses (on the order of nanoseconds). These laser pulses cause desorption and ionization of analyte molecules in a complex multistep process.^6,7^ Moreover, MALDI does not require labeling, thus preserving the native chemical composition of the sample.

In MALDI MSI, the tissue surface is scanned in a rasterized manner, and a mass spectrum is acquired for each sampled position (pixel). After data acquisition, ion distribution images for selected *m*/*z* values are reconstructed, where pixel intensity reflects ion abundance (Figure 1A). These images visualize the spatial localization of compounds within the tissue.^2,3^

**Figure 1:**
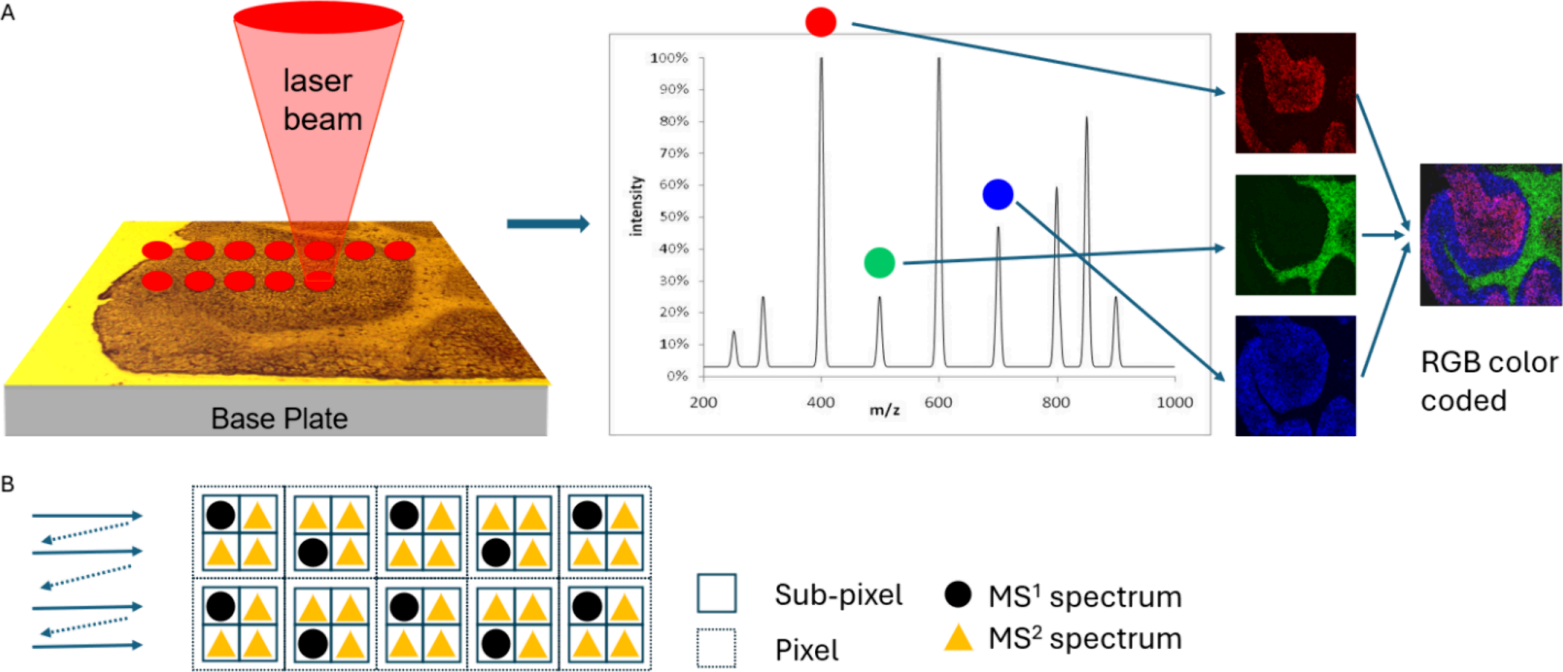
Scheme of the data acquisition strategy for MALDI MSI in general and as applied in this study. (A) The matrix-coated sample is rasterized with a laser beam; the ionized analytes are detected, producing one mass spectrum per ablated spot. Ion distribution images for selected *m*/*z* values are generated, and composite images can be created by combining multiple ion images in RGB channels. (B) In this study, the sample is rasterized in pixel mode from left to right while acquiring alternatingly one MS^1^ and three MS^2^ spectra. Each acquired spectrum corresponds to one subpixel, resulting in a 2 × 2 array of subpixels that form a pixel in the final MS image.

Typical MALDI MSI spot sizes range from 50 μm down to a few micrometers, enabling subcellular spatial resolution.^5^

Historically, MSI data acquisition has focused on MS^1^ (full scan) data, where compound annotation is based mainly on accurate precursor-ion *m*/*z* values.^8–10^ However, this approach often leads to ambiguous annotations, because multiple components have the same or nearly identical *m*/*z* values.^9^ MS^2^ (MS/MS) spectra can provide complementary information that can increase annotation confidence through comparison with known spectra in databases or by denovo analysis.^11^ Moreover, fragment ions may additionally support discrimination of structurally related compounds or lipid classes.^10^

Several targeted MS^2^ imaging approaches have been developed where predefined precursor ions are fragmented to generate MS^2^ ion images.^12–14^ Comparing MS images from precursor ions with their MS^2^ fragment images enables confirmation of spatial distributions for compounds with similar masses but different fragmentation behaviors. MS^2^ imaging has also been applied to identify the spatial distribution of various phospholipid classes, each characterized by specific diagnostic fragments.^15^

Data-dependent acquisition (DDA) has also been integrated into MSI workflows. In DDA, precursor ions are dynamically selected for fragmentation based on real-time analysis of MS^1^ data.^16^ Although MS^2^ spectra are not typically used for MS^2^ image generation, they significantly enhance compound identification reliability by matching MS^2^ spectra to spectral libraries.^16^ Dynamic exclusion lists help to maximize coverage by avoiding repeated fragmentation of the same precursor ions within a certain time window. Nonetheless, DDA tends to select the most intense precursor ions detected in each MS^1^ spectrum, potentially overlooking lower-intensity compounds even when they are spatially localized within the sample.

Additionally, a data set-dependent approach was introduced which first acquires MS^1^ imaging data, analyzes the MS^1^ data to identify precursor ions of interest and their spatial distribution and then acquires MS^2^ data from the same sample region based on this MS^1^ information.^17^

In liquid chromatography–mass spectrometry (LCMS), data-independent acquisition (DIA) has gained increasing popularity, particularly in proteomics. DIA allows fragmentation of all ions within sequential *m*/*z* windows, providing unbiased and comprehensive coverage of the entire mass range. Compared to DDA, DIA offers higher reproducibility and better detection of low-abundance species, since no ions are preferentially selected. However, data analysis is more complex because DIA typically uses larger *m*/*z* windows, resulting in spectra that contain fragment ions from multiple compounds, making interpretation challenging.^18^

One implementation that combines the DIA principle with MSI is Waters’ high-definition mass spectrometry (HDMS^E^), which combines ion mobility separation (IMS) with DIA. In this approach, the instrument alternates rapidly between low-energy scans that record accurate precursor ions and elevated-energy collision-induced dissociation (CID) scans that generate fragment ions. The resulting ions are aligned according to their ion mobility drift times, allowing accurate correlation between precursor and fragment ions. This drift-time alignment enables the reconstruction of comprehensive spectral information for each compound.^19,20^

Here, we introduce a MALDI MSI workflow integrating DIA to combine spatially resolved MS^1^ imaging with complementary MS^2^ fragmentation information. MS^1^ data are used to create MS images, while DIA MS^2^ data support compound annotation. To reduce spectral complexity, relatively narrow DIA *m*/*z* windows are used. DIA window order is randomized across the tissue to minimize systematic sampling bias and improve spatial sampling of different *m*/*z* regions. The presented workflow does not perform full computational deconvolution of DIA spectra. Instead, precursor alignment is constrained through narrow isolation windows, spatial correlation with MS^1^ precursor-ion distributions, and selection of spectra acquired at positions with high precursor abundance and low coisolation interference.

For data processing, we use an extended version of Thermo Scientific Compound Discoverer (CD). This software is designed for the analysis and identification of small molecules in complex samples using high-resolution mass spectrometry data. It provides a comprehensive suite of tools for processing, analyzing, and interpreting mass spectrometry data to identify and quantify compounds. CD is widely used in various fields, including metabolomics, pharmaceutical research, environmental analysis, and food testing.^21^

Among its features, CD provides tools for the identification of unknown compounds, such as Thermo Scientific LipidSearch^22^ and Thermo Scientific mzCloud (https://www.mzcloud.org/), which are integrated as processing nodes. LipidSearch enables lipid identification based on accurate MS^1^ *m*/*z* values and uses theoretical MS^2^ fragmentation patterns to accurately identify lipid species and their structural components. mzCloud is an online mass spectral database containing high-quality mass spectra curated by experts for various compounds. It matches experimental spectra with those in the high quality mzCloud library to identify compounds.

To demonstrate the workflow, we analyzed samples of *Fasciola hepatica*, commonly known as the liver fluke, which is a parasitic flatworm infecting the livers and bile ducts of mammals such as humans, cattle and sheep. This parasite is responsible for the disease known as fascioliasis.^23^ Previous AP-SMALDI MSI studies investigated the spatial distribution of therapeutic compounds in *F. hepatica*.^24,25^ In this work, DIA fragmentation information was additionally used to support spatially resolved annotation of endogenous lipids and the model compound imatinib directly from the tissue section.

## Methods/Experiments

### Sample Preparation

#### Experimental Animals and Parasites

Rats (*Rattus norvegicus*) were used as model hosts in accordance with the European Convention for the Protection of Vertebrate Animals used for Experimental and Other Scientific Purposes (ETS No 123; revised Appendix A). All animal experiments were conducted at the Institute of Parasitology, Justus Liebig University Giessen, and were approved by the Regional Council (Regierungspraesidium) Giessen (V54–19c20 15 h 02 GI 18/10 Nr. A16/2018).

Male Wistar rats RjHan:WI, *Rattus norvegicus* (Janvier, France) served as hosts to cultivate adult *Fasciola hepatica*. At 5 weeks old, the rats were orally administered 25 metacercaria from an Italian parasite strain (Ridgeway Research, UK). Adult flukes were collected from the bile ducts 12 weeks after infection. Prior to use in experiments, the worms were kept in 0.9% NaCl for at least 1 h to ensure the clearance of gut contents.

#### Drug Treatment and Preparation of *F. hepatica* for AP-SMALDI MSI

Adult *Fasciola hepatica* were exposed to the ABLTK inhibitor imatinib [imatinib mesylate, purity ≥ 98% (HPLC); Enzo Life Sciences, Germany] for 4 h. The drug was administered at 100 μM in RPMI medium, supplemented with 5% chicken serum and 1% ABAM solution [10,000 units penicillin, 10 mg streptomycin, and 25 mg amphotericin B per ml] (all from Sigma-Aldrich, Gibco). Following the drug treatment, the worms were thoroughly washed in PBS solution and then in distilled water. They were subsequently placed in a Tissue-Tek Cryomold (15 × 15 × 5 mm, Sakura Finetek, Netherlands) containing an 8 wt % liquid aqueous gelatin solution (gelatin powder, VWR, USA) and frozen on dry ice. The worm samples were stored at −80 °C until further use.

Transversal sections (20-μm thickness) of the embedded *F. hepatica* were prepared in a cryostat HM525 (Thermo Fisher Scientific) at around −23 °C. Section quality was monitored using a digital light microscope (VHX-5000, Keyence, Japan). After quality control, the sections were stored at −80 °C until AP-SMALDI MSI analysis.

Before applying the matrix, the worm sections were thawed and placed in a desiccator for 20 min to protect them from humidity. A matrix solution of 2,5-dihydroxybenzoic acid (DHB, for synthesis, Merck, Germany) was prepared at a concentration of 30 g/L in a 1:1 v/v mixture of acetone (acetone uvasol, Merck) and water (HiPerSolv Chromanorm for HPLC, filtered at 0.2 μm, VWR), with the addition of 0.1 vol % trifluoroacetic acid (TFA, uvasol for spectroscopy, Merck). 80 μL of the DHB solution was sprayed onto the section surface using an ultrafine pneumatic sprayer system (SMALDIPrep, TransMIT GmbH, Giessen, Germany) at a flow rate of 10 μL/min and an N_2_ pressure of 1 bar.

### Data Acquisition

The DIA-specific data acquisition method is described in detail in the Results section.

MALDI MSI was performed on a Thermo Scientific Orbitrap Exploris 480 mass spectrometer (Thermo Fisher Scientific) equipped with an autofocusing AP-SMALDI^5^ AF ion source (TransMIT GmbH) that operates under atmospheric pressure.^26^ The ion source was operated in 2D pixel mode with a step size of 10 μm and 50 laser pulses per pixel. In general, data acquisition consists of alternating one MS^1^ scan with three DIA MS^2^ scans. All experiments were performed in positive-ion mode. For the MS^1^ experiment a mass resolution of 240,000 at *m*/*z* 200 and an *m*/*z* range of 250–1,000 were chosen. For internal mass locking, Easy-IC was used scan-to-scan.

For the DIA experiment a mass resolution of 45,000 at *m*/*z* 200 and a normalized collision energy of 30% was applied. A user-defined DIA *m*/*z* window list covering *m*/*z* from 650 to 899 Da with an isolation width of 3 Da per window was used. In addition, one dedicated DIA isolation window covering the imatinib precursor ion (*m*/*z* 492.5–495.5) was included in the acquisition list. The loop control was configured to acquire one MS^1^ scan followed by three DIA MS^2^ scans, corresponding to a loop count of three spectra. During acquisition, the DIA windows were executed cyclically across the tissue section, resulting in neighboring image pixels sampling different DIA windows.

A maximum ion injection time of 500 ms, an S-lens RF level of 50 arbitrary units, a capillary temperature of 250 °C, and an acceleration voltage of 3.5 kV were specified. Data acquisition was controlled using OES 4.4 Instrument Control software, version 4.2 SP2, and SMALDI control software, version 1.6.

A custom tool was developed to generate randomized DIA *m*/*z* window lists. The tool takes as input the total *m*/*z* range, isolation width, and maximum number of windows. All possible nonoverlapping *m*/*z* intervals are calculated, repeatedly randomized, and subsequently truncated to the desired number of windows if necessary.

### Data Analysis

After data acquisition, spectra were processed using Freestyle 1.8 SP2 (Thermo Fisher Scientific) and an extended version of Compound Discoverer 3.4 (Thermo Fisher Scientific). Compound annotation was performed using mzCloud, LipidSearch (both Thermo Fisher Scientific) and the LIPID MAPS database.^27^ DIA MS^2^ spectra were associated with precursor candidates based on isolation-window information and spatial correlation with MS^1^ precursor-ion distributions prior to spectral-library matching.

## Results

### Overview of the Data Acquisition Method

In this work, we adopt terminology analogous to display technology: an image is composed of multiple pixels, and each pixel is made up of several subpixels. In our context, one mass spectrum is acquired for each subpixel: one is a full MS^1^ spectrum, and the others are MS^2^ spectra positioned around the MS^1^ spectrum. All subpixels share the same spatial resolution. The overall spatial resolution of a pixel is therefore determined by both the number of subpixels in the *x*- and *y*-directions and the spatial resolution of individual subpixels. Depending on the arrangement of the subpixels, pixels can be either rectangular (e.g., 1 × 3 subpixels) or square (e.g., 2 × 2 subpixels).

In our approach, a selected region of interest within the sample is scanned by alternating one MS^1^ spectrum with *n* MS^2^ spectra (Figure 1B). The number of MS^2^ spectra collected between two consecutive MS^1^ spectra is chosen such that, during image reconstruction, each image pixel contains one MS^1^ spectrum and results in a square pixel geometry. MS^1^ and MS^2^ spectra are acquired within a single raw file acquisition without prior knowledge of compound locations. The MS^2^ spectra are acquired in a data-independent manner (DIA MS^2^), meaning that all ions within predefined *m*/*z* windows are fragmented simultaneously, ensuring comprehensive coverage of all detectable ions.

The total number of DIA *m*/*z* windows depends on the *m*/*z* range of interest and the chosen isolation width. In most cases, the total number of windows greatly exceeds the number of subpixels per image pixel, so that only a subset of *m*/*z* windows is sampled within each reconstructed pixel. The desired pixel resolution of the MS^1^ image determines the ratio between MS^1^ and MS^2^ acquisitions. Optimization of the acquisition strategy therefore requires balancing spatial sampling density, total mass range coverage, and isolation window width.

To minimize systematic sampling effects arising from regular scan patterns and to increase the probability of sampling different *m*/*z* regions throughout the tissue, DIA acquisition positions are randomly distributed across the region of interest.

After data acquisition, ion images are constructed from the MS^1^ spectra to visualize the spatial compound distributions, while DIA MS^2^ spectra are used to support compound annotation. Typically, DDA-based compound annotation tools require precursor-ion information for MS^2^ spectra to restrict the search space. In DDA experiments, this is derived from the parent MS^1^ spectrum. DIA MS^2^ spectra do not contain explicit precursor assignment. In the presented workflow, precursor candidates are inferred from the MS^1^ spectrum within each reconstructed image pixel together with DIA isolation window information. This approach enables precursor associated DIA MS^2^ annotation while maintaining spatial integrity.

The interdependence of DIA *m*/*z* window design and spatial sampling is illustrated in Figure 2. Increasing the DIA *m*/*z* window width reduces the total number of windows required to cover a defined mass range but increases spectral complexity due to enhanced cofragmentation (Figure 2A). Alternatively, extending mass-range coverage by increasing the number of DIA *m*/*z* windows while maintaining constant window width increases the spatial distance between repeated acquisitions of the same *m*/*z* window, thereby reducing the likelihood of fragmenting precursor ions at their most relevant spatial locations, particularly when analytes are confined to small regions (Figure 2B). Increasing the number of subpixels per reconstructed pixel allows sampling of more DIA *m*/*z* windows within each reconstructed pixel. However, this also increases MS^1^ spacing and consequently lowers the effective MS^1^ spatial resolution (Figure 2C). Together, these relationships demonstrate that DIA *m*/*z* window configuration directly influences spectral complexity, spatial sampling frequency and effective image resolution.

**Figure 2:**
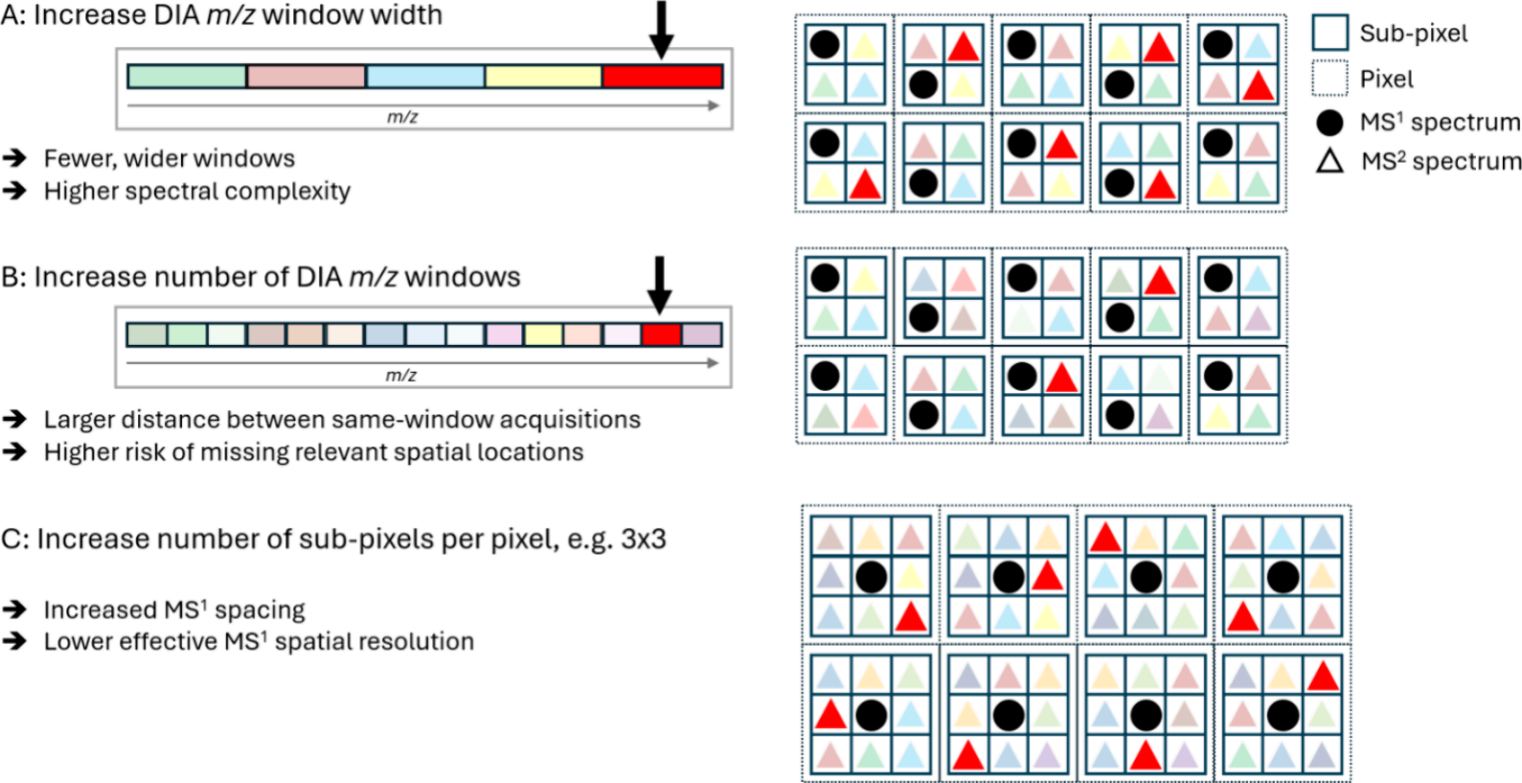
Schematic representation of trade-offs in the proposed DIA MSI acquisition strategy. (A) Increasing DIA *m*/*z* window width reduces the number of isolation windows required for a given mass range but increases spectral complexity due to cofragmentation. (B) Increasing the number of DIA *m*/*z* windows extends mass-range coverage at constant window width but increases the spatial distance between repeated acquisitions of the same window, thereby increasing the risk of missing relevant spatial locations of precursor ions. (C) Increasing the number of subpixels per pixel (e.g., 3 × 3) enables sampling of more DIA windows per reconstructed pixel but increases MS^1^ spacing and reduces effective MS^1^ spatial resolution.

### Compound Discoverer Workflow

Following data acquisition, the MSI data sets were processed using a customized workflow implemented in CD. CD is primarily designed for the analysis of Liquid Chromatography–Mass Spectrometry (LC-MS) data, where compounds are separated chromatographically prior to mass spectrometric detection. In LC-MS experiments, compounds elute from the chromatographic column at characteristic retention times, producing elution peaks that facilitate compound detection and alignment.

In contrast, MSI approaches do not contain chromatographic separation or retention-time information. Instead, spectra are acquired sequentially from spatial positions across the sample surface, such that acquisition order reflects spatial location rather than chromatographic elution. Standard processing pipelines in CD are therefore not directly applicable to MSI data and were extended here with custom processing nodes supporting MSI-specific data handling and DIA MS^2^ analysis.

The workflow first assigns each acquired mass spectrum to its spatial location within the MSI data set using image-acquisition metadata from the position information file (.udp) generated by the SMALDI control software. Based on these spatial coordinates, MS^1^ spectra are analyzed to identify relevant *m*/*z* ranges and reconstruct corresponding MS images.

LipidSearch and mzCloud are integrated into the CD framework and operate in a spectrum-centric manner independent of retention-time information. Both tools compare MS^2^ spectra with theoretical fragmentation patterns or spectral libraries and were adapted here for precursor-associated DIA MS^2^ analysis. For this study, the LipidSearch configuration was additionally extended to include K^+^ adducts, which were abundant in the MSI data set.

Because DIA MS^2^ spectra are associated with isolation windows rather than individual precursor ions, an additional precursor-assignment step was required prior to annotation. The presented workflow does not perform full computational deconvolution of DIA spectra. Instead, precursor candidates are associated with DIA MS^2^ spectra using MS^1^ precursor-ion distributions together with matching DIA isolation window information. To improve annotation confidence and reduce coisolation interference, DIA MS^2^ spectra acquired at positions with high precursor abundance and low spectral interference were preferentially selected for spectral-library matching. This quality-controlled spectral selection was applied only during data analysis, whereas DIA acquisition itself remained unbiased across the tissue section.

Finally, annotation results from LipidSearch and mzCloud were linked to the corresponding precursor-ion images. Lipid annotations detected though different adduct forms were grouped and evaluated using Pearson correlation of their spatial distributions.^28^ Pearson correlation values >0.7 indicated good agreement between adduct images and provided additional support for lipid-group annotations.

An additional processing node was implemented for spectrum-centric analysis of raw MSI data independent of image generation. This node extracts user-defined *m*/*z* values from MS^1^ or MS^2^ spectra and determines the corresponding mass deviations (in ppm and Da) relative to specified target values. The resulting tabulated output was used for assessment of mass accuracy and spectral stability throughout the acquisition.

MS images were visualized either as single-channel intensity images or using the *viridis* false-color colormap. For all visualizations, ion intensities were scaled to a defined display range to facilitate comparison of spatial intensity distributions across tissue sections.

### Raw File Acquisition and Quality Assessment

The *F. hepatica* sample was rasterized with 230 × 686 subpixels, where each reconstructed pixel consisted of 2 × 2 subpixels, resulting in a final image raster of 115 × 343 pixels. The step size (subpixel size) was 10 μm in both x- and *y*-directions resulting in a rasterized sample area of 2.3 × 6.8 mm^2^ and an effective image resolution of 20 × 20 μm per pixel.

For the DIA acquisition, a randomized *m*/*z* range list was used, comprising 83 *m*/*z* ranges spanning *m*/*z* 650–899 Da, each with a 3 Da isolation width. An additional isolation window of *m*/*z* 492.5–495.5 Da was included for acquisition of imatinib-related DIA MS^2^ spectra. Thus, both the imatinib and lipid analyses presented in this study were derived from the same MSI data set acquired in a single measurement. The resulting raw file had a size of 2.2 GB and contained approximately 158,000 spectra (39 k MS^1^, 118 k DIA MS^2^), with each DIA window acquired approximately 1,400 times throughout the data set.

The total data acquisition time was approximately 30 h. Given this extended duration, mass accuracy and temporal stability of both MS^1^ and MS^2^ data were evaluated. MS^1^ mass accuracy was estimated by calculating the mass deviation of common DHB cluster ions, as described previously.^29^ Figure 3A shows the mass deviations of two abundant DHB cluster ions (3M+NH_4_-2H_2_O^+^: *m*/*z* 444.09252, 6M+NH_4_-5H_2_O^+^: *m*/*z* 852.14065). Mass deviations remained stable throughout the acquisition without observable drift. Based on these findings, the precursor mass tolerance for LipidSearch, mzCloud, and MS image generation was set to ± 3 ppm.

**Figure 3:**
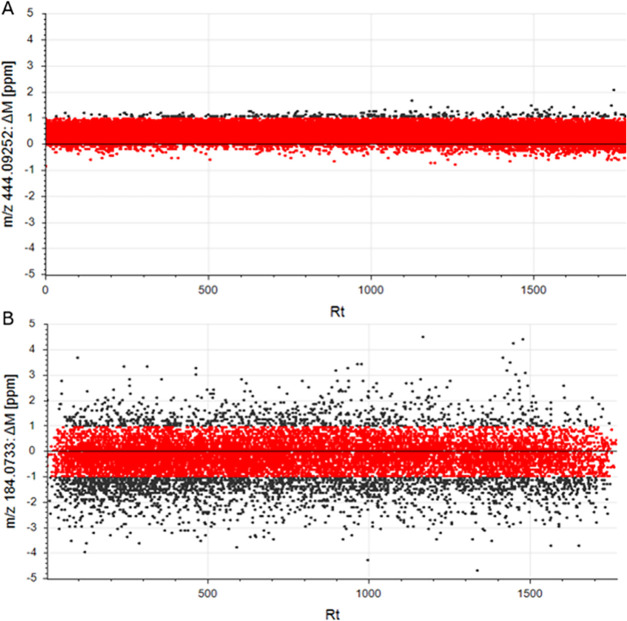
Mass deviation plots of (A) the DHB clusters at *m*/*z* 444.09252 in MS^1^ spectra, and (B) the phosphocholine fragment at *m*/*z* 184.0733 in MS^2^ spectra. Mass deviation (Δ*M*ass, ppm) is shown as a function of acquisition time. Red points indicate deviations within ± 1 ppm. MS^1^ and MS^2^ mass accuracies remained within approximately ±2 and ±4 ppm, respectively, without observable drift.

MS^2^ mass accuracy was evaluated using common DHB fragment ions as well as fragment ions of annotated compounds, including imatinib and lipids. In this data set, MS^2^ fragment-ion intensities were generally lower than those observed in MS^1^ spectra, resulting in only a limited number of fragment ions being detected consistently. The dehydrated DHB-cluster ion (*m*/*z* 273.04) and the phosphocholine fragment (*m*/*z* 184.0733) which represents the headgroup of phosphatidylcholine (PC) lipids were frequently detected. Figure 3B displays the corresponding mass deviations, which remained within ± 4 ppm throughout the acquisition without evidence of temporal drift. Accordingly, the fragment mass tolerance for data processing was set to ± 5 ppm.

### DIA MS^2^-Based Identification of Imatinib

To illustrate the DIA acqusition and precursor-assignment strategy, the imatinib precursor ion detected within the MSI data set was used as a model compound. Figure 4A displays an optical image of the transversal *F. hepatica* section taken prior to MSI analysis, with major anatomical structures annotated. Figure 4B shows the MS image of *m*/*z* 494.2666, corresponding to [M + H]^+^ ion of imatinib. The MS image reveals that imatinib was distributed throughout the tissue, with the highest signal intensities observed in the intestinal and vitellarium tissue.

**Figure 4:**
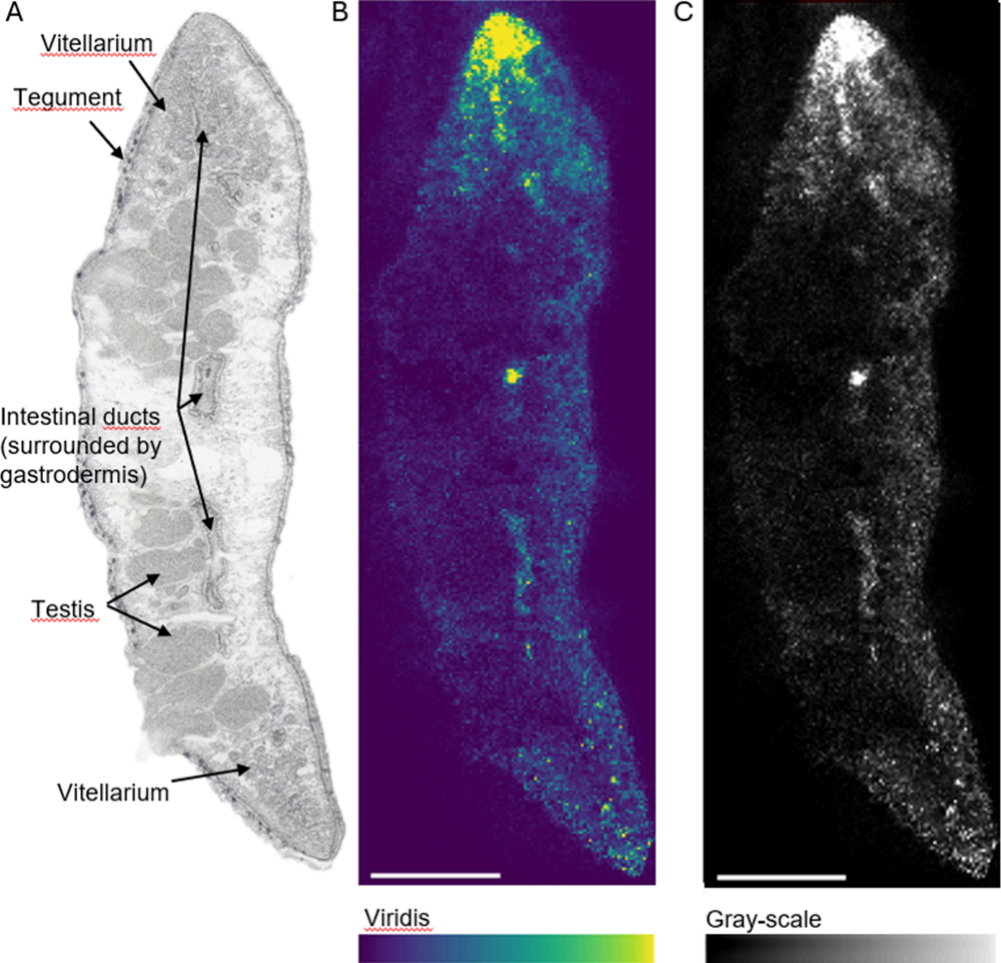
Tissue context and spatial distribution of imatinib in an *F. hepatica* tissue section. (A) Optical image of the analyzed *F. hepatica* tissue with major anatomical structures indicated. (B, C) MS images showing the spatial distribution of the imatinib precursor ion at *m*/*z* 494.2666 [M + H]^+^, displayed using the viridis color palette (B) and a grayscale intensity scale (C). Imatinib is spread throughout the body with the highest signal intensities observed in the intestinal and vitellarium tissue. Scale bars correspond to 1 mm.

Figure 5A shows the spatial distribution of the imatinib precursor ion together with the positions at which DIA MS^2^ spectra were acquired for the isolation window *m*/*z* 492.5–495.5. This isolation window includes the [M + H]^+^ precursor ion of imatinib and therefore enables acquisition of corresponding fragment-ion information where the precursor is present. The regular acquisition patterns result from cyclic execution of the DIA *m*/*z* window list during data acquisition. The figure demonstrates that DIA MS^2^ spectra covering the imatinib precursor window were acquired at multiple tissue locations with evaluated precursor ion intensity.

**Figure 5:**
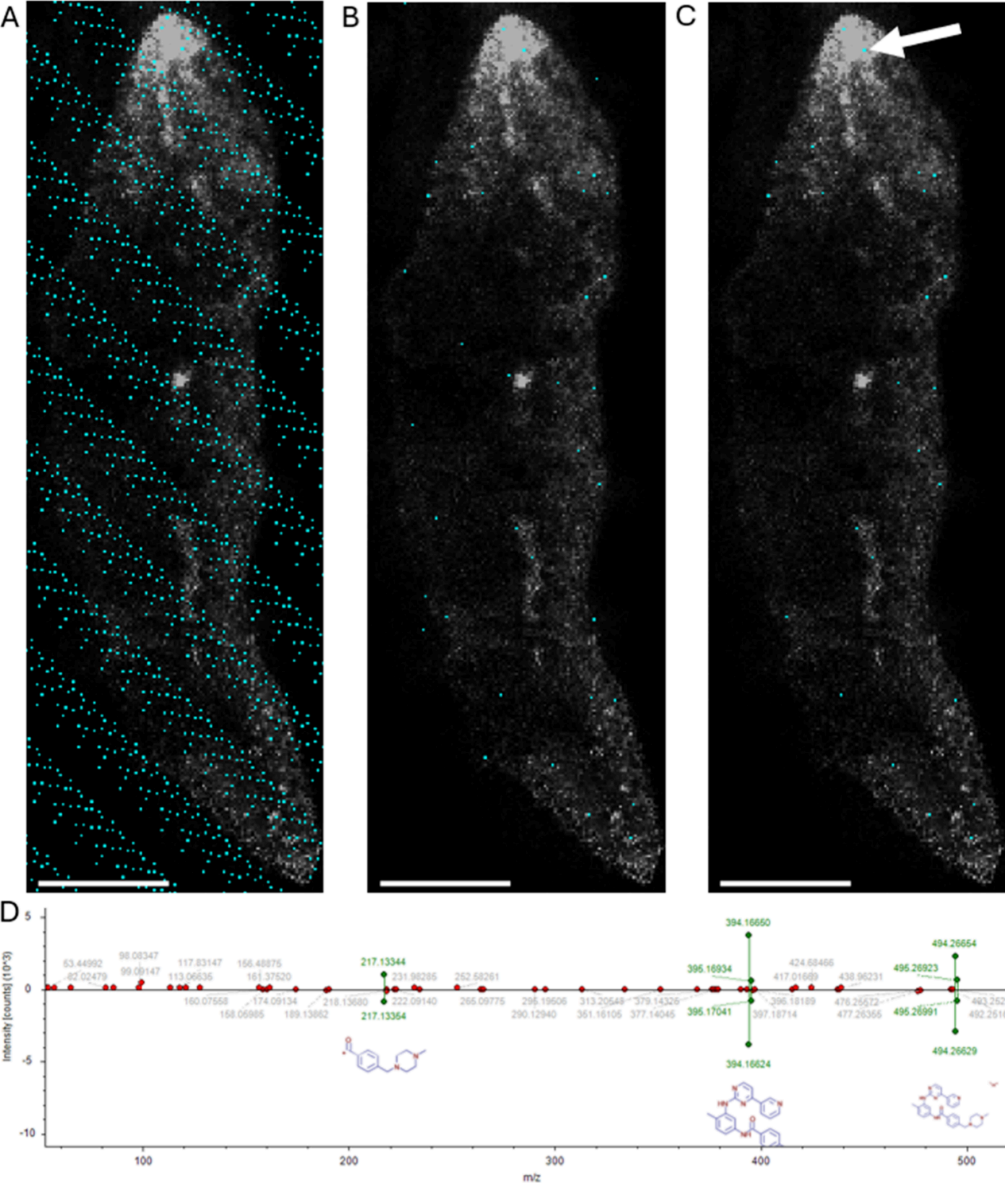
Spatial correlation of DIA MS^2^ acquisition spots with imatinib precursor-ion distribution. (A–C) Grayscale MS images of the imatinib precursor ion (*m*/*z* 494.2666) with overlaid DIA MS^2^ acquisition spots shown in cyan, corresponding to all spots within the isolation window 492.5–495.5 (A), the subset selected for mzCloud spectral library matching (B), and the further subset annotated as imatinib by mzCloud (C). Precursor-ion intensities were scaled to 70% of the maximum intensity to improve contrast with overlaid acquisition positions. The arrow in panel (C) indicates the acquisition spot used for MS^2^ spectral comparison in panel (D). Scale bars correspond to 1 mm. (D) Representative DIA MS^2^ spectrum acquired from the highlighted spot in panel (C), shown as a mirror plot together with the matched mzCloud library spectrum. Peaks shared between the experimental and library spectra are shown in green, while unmatched peaks are shown in red.

Figure 5B shows the subset of DIA MS^2^ acquisition spots that were submitted to an mzCloud spectral-library search. To reduce mzCloud processing time and minimize spectral interference, only a selected subset of DIA MS^2^ spectra acquired at positions with relatively high precursor-ion abundance and low coisolation interference was selected for annotation. The selected spectra additionally maintained representative spatial sampling across the tissue section.

Figure 5C displays the further subset of acquisition spots for which DIA MS^2^ spectra were annotated as imatinib by mzCloud. Annotations were obtained only at locations where the imatinib precursor ion was present within the isolation window, whereas spectra acquired at positions lacking detectable precursor signal could not be annotated.

Figure 5D shows a representative DIA MS^2^ spectrum acquired from the highlighted spot in Figure 5C, displayed as mirror plot together with the matched mzCloud library spectrum. Fragment ions shared between the experimental and library spectra are highlighted in green. The close agreement of dominant fragment ions supports the precursor-associated annotation of imatinib and demonstrates the feasibility of integrating DIA fragmentation information with MSI data. The imatinib experiment primarily serves as a proof-of-principle demonstration of the DIA workflow and precursor-association strategy. Following validation of the DIA MS^2^ MSI approach using imatinib, subsequent analyses focused on endogenous lipid annotations.

### DIA MS^2^-Based Identification of Lipids

mzCloud and LipidSearch were evaluated for lipid annotation. mzCloud annotated only a single lipid group (PC 32:0, [M + H]^+^) due to the limited number of lipid MS/MS spectra currently available in the mzCloud library. Therefore, subsequent analyses focused on LipidSearch.

LipidSearch supports the annotation of several lipid classes, including phosphatidylcholine (PC), phosphatidylethanolamine (PE), and hexosylceramide (HexCer). Integrated as a processing node in CD, LipidSearch performs searches based on accurate precursor-ion mass and can additionally incorporate MS^2^ spectral information. In LipidSearch nomenclature, a lipid group is defined by lipid class and total acyl carbon number and unsaturation, without differentiation of sn-positions or double-bond positional isomers.

Using a precursor tolerance of 3 ppm, LipidSearch returned 550 lipid groups based on precursor-ion mass alone. When DIA MS^2^ spectra were included, this number was reduced to 393 lipid groups, indicating that approximately 150 candidate annotations were excluded based on fragment-ion information from the DIA MS^2^ spectra. Of the excluded lipid groups, 100 were PE species for which the expected neutral-loss fragment of 141.0191 Da from the precursor ion^30^ was not detected in the DIA MS^2^ spectra. Additionally, 19 PC and 19 HexCer annotations were excluded because the corresponding DIA MS^2^ spectra lacked the expected phosphate headgroup fragment or other characteristic fragment ions.

Because LipidSearch returned a high number of candidate lipid annotations, additional filtering based on MSI-derived spatial information was applied to reduce false positives. MS images assigned to the same lipid but detected through different adducts are expected to show similar spatial intensity distributions, as they originate from the same underlying compound. This similarity was assessed using the Pearson correlation coefficient. In addition, consistency checks across DIA MS^2^ spectra acquired from the same isolation window were used to reduce random spectral matches.

When expected adduct forms were detected, corresponding MS images showed high spatial similarity, and multiple DIA MS^2^ spectra acquired at positions with high precursor-ion intensity and low coisolation interference supported the same lipid-group annotation. Conversely, annotations were excluded when expected adduct images showed inconsistent spatial distribution or lacked supporting fragment-ion information.

Applying combined precursor mass, DIA MS^2^, and spatial filtering reduced the initial list of 393 lipid groups to nine phospholipid annotations. Figure 6 shows MS images of PC 32:0 detected as the [M + H]^+^, [M + Na]^+^, and [M+K]^+^ adducts, which exhibit highly similar spatial distributions (Pearson correlation coefficient = 0.86). DIA MS^2^ spectra acquired from corresponding spatial locations contained characteristic phosphatidylcholine fragment ions, thereby providing additional support for the lipid-group annotation across all adduct forms. MS images of all nine confidently annotated lipid groups are shown in Figure 7.

**Figure 6:**
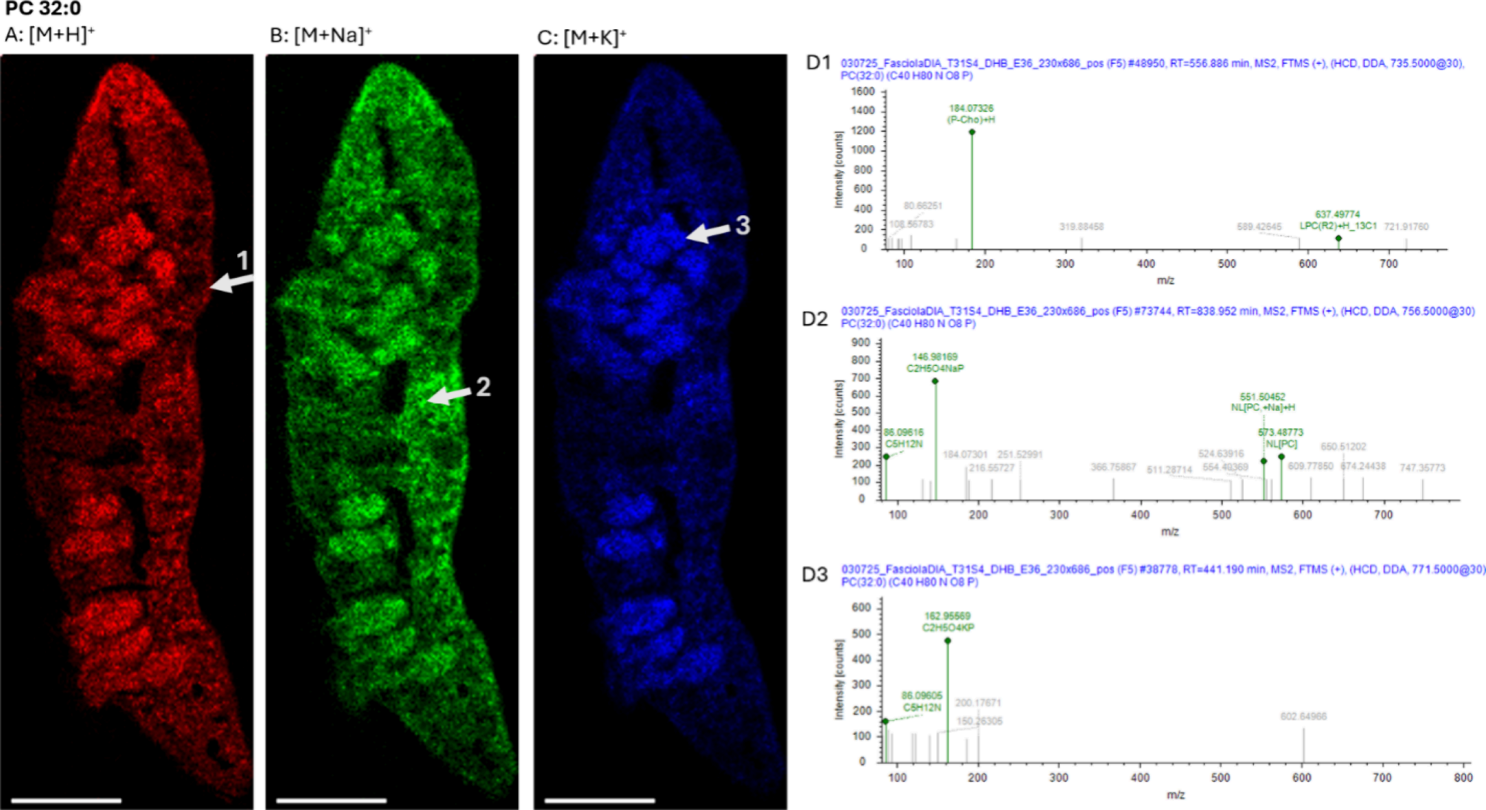
MS images and corresponding DIA MS^2^ spectra of PC 32:0 detected in *F. hepatica* tissue. (A–C) MS images showing the spatial distribution of PC 32:0 detected as different adduct forms: [M + H]^+^ (A), [M + Na]^+^ (B), and [M+K]^+^ (C). All three adducts exhibit highly similar spatial distributions across the tissue section, supporting their assignment to the same lipid group. Arrows indicate representative spatial locations from which DIA MS^2^ spectra were acquired. (D1–D4) Representative DIA MS^2^ spectra acquired from the highlighted locations in panels (A)–(C), showing characteristic phosphatidylcholine fragment ions. Scale bars correspond to 1 mm.

**Figure 7:**
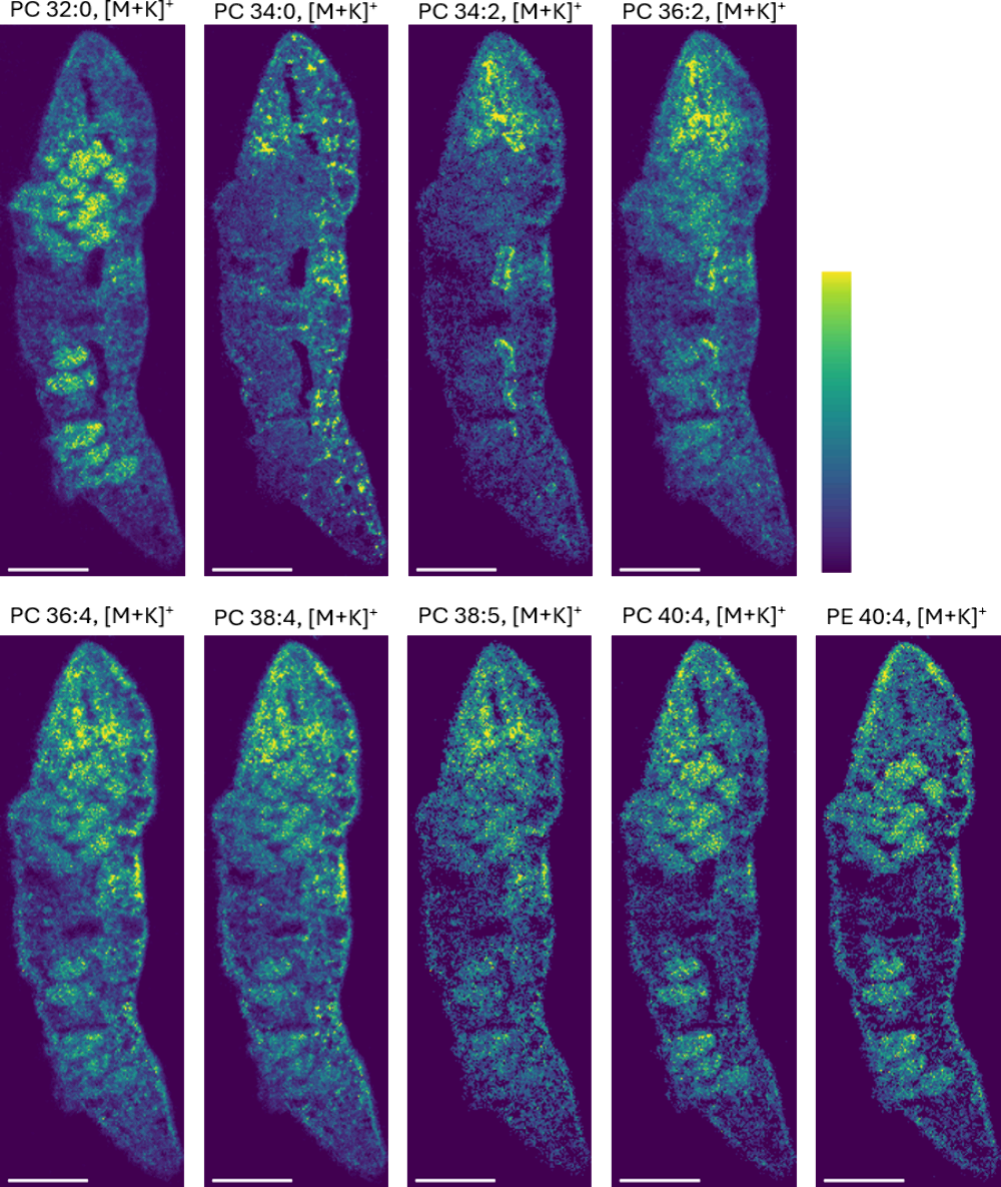
MS images of the nine confidently annotated lipid groups detected in *F. hepatica* tissue. MS images show the spatial distribution of the nine phospholipid groups retained after combined precursor mass, DIA MS^2^, and spatial filtering. Lipid group annotations and adduct forms are indicated above each panel. Images are displayed using the viridis color palette. Scale bars correspond to 1 mm.

Several MS images were not annotated by LipidSearch due to limitations of the configured lipid class definitions. Two groups of MS images showed high spatial similarity and precursor mass differences consistent with adduct exchange, indicating detection of the same underlying compounds with different adduct forms. One group was localized to the tegument of *F. hepatica*, while the other was confined to the intestinal ducts. Both compounds were detected with mass offsets corresponding to [M + H]^+^, [M + Na]^+^, and [M+K]^+^ adducts.

These compounds were restricted to small, localized tissue regions, resulting in only a limited number of pixels with sufficient precursor-ion intensity for DIA MS^2^ acquisition. In these regions, DIA MS^2^ spectra were acquired but remained dominated by the precursor-ion signal, indicating limited fragmentation efficiency. Manual annotation using the LIPID MAPS database suggested HexCer 38:0;O4 and HexCer 36:0;O3 for the two image groups; however, these assignments remain tentative, because the observed fragmentation and adduct patterns did not fully confirm the proposed structures. Representative MS images and corresponding DIA MS^2^ spectra of the sodium adducts are shown in Figure 8.

**Figure 8:**
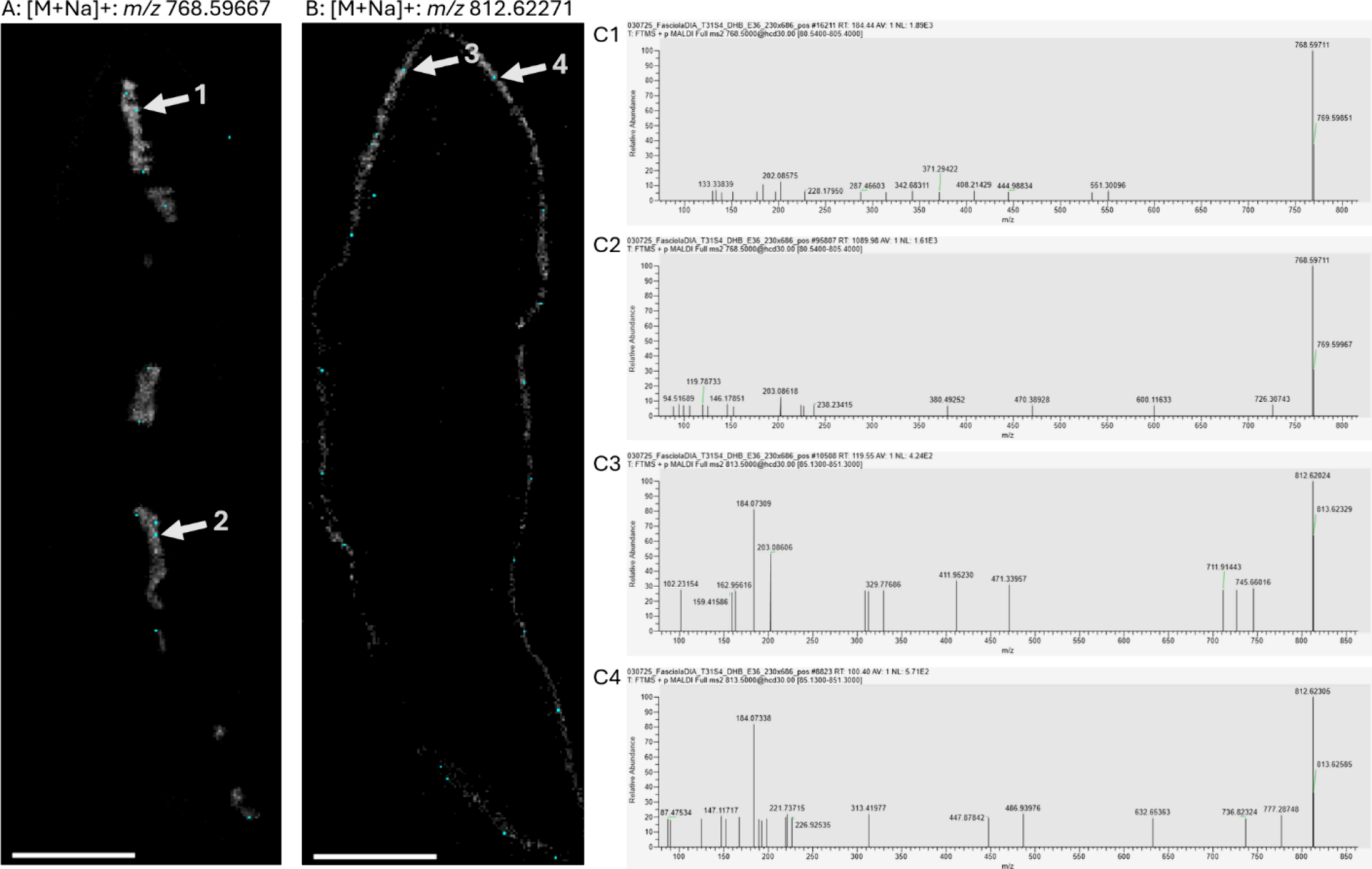
Spatial correlation of DIA MS^2^ acquisition spots with the distribution of two unannotated compounds detected as sodium adducts in F. hepatica tissue. (A, B) Grayscale MS images showing the spatial distributions of two unannotated compounds detected as [M + Na]^+^ adducts at *m*/*z* 768.59667 (A) and *m*/*z* 812.62271 (B), with overlaid DIA MS^2^ acquisition spots shown in cyan. Cyan pixels indicate spatial locations where DIA MS^2^ spectra of sufficient quality were acquired within isolation windows encompassing the corresponding precursor *m*/*z* values and were selected for annotation analysis. These locations are spatially correlated with the presence of the respective precursor ions. (C1–C4) Representative DIA MS^2^ spectra acquired from the arrow-indicated locations in panels (A) and (B). The spectra are dominated by precursor-ion signals and exhibit limited fragmentation. Scale bars correspond to 1 mm.

## Discussion

Combining MSI with DIA MS^2^ proved to be an effective strategy for integrating spatially resolved MS^1^ imaging with complementary fragmentation information. The method enables acquisition of fragment-ion data across the entire investigated mass range, thereby reducing the bias toward highly abundant precursors characteristic of DDA strategies. As a result, low-abundant species that might be overlooked in DDA workflows are more consistently fragmented and recorded. Additionally, randomization of DIA isolation windows improves spatial sampling robustness compared with fixed sequential acquisition schemes. The comprehensive nature of DIA data sets also permits retrospective analysis, allowing compound annotations to be re-evaluated or refined without additional measurements.

Despite these advantages, several analytical limitations must be considered. One limitation of the DIA approach is the use of relatively large isolation windows compared with DDA, which increases precursor coisolation and consequently spectral complexity. This issue is particularly relevant for lipid analysis, where structurally related lipid species may overlap within the same isolation window. Although narrower isolation windows improve spectral selectivity, they reduce spatial sampling efficiency. Consequently, the number and width of DIA windows must be carefully optimized to balance spectral specificity against spatial coverage. Integration of complementary MS^1^ information can further support precursor assignment and mitigate ambiguities arising from coisolation.

Importantly, the presented workflow does not perform full computational deconvolution of DIA spectra and therefore does not provide unambiguous structural elucidation for all compounds, particularly positional isomers and isobaric lipid species. Instead, the workflow improves annotation confidence through integration of precursor mass, spatial correlation, DIA fragmentation, and spectral-library matching.

A specific example illustrating these limitations concerns the apparent exclusion of PE lipids in the present study. Several PE lipid annotations were not supported by DIA MS^2^ because the characteristic neutral-loss fragment of 141.0191 Da was not observed in the acquired spectra. However, the absence of this diagnostic fragment should not be interpreted as definitive evidence that the corresponding PE species are absent. Instead, it likely reflects the lower ionization and detection sensitivity of PE lipids compared with PC lipids. Therefore, caution is required when interpreting MS^2^-based lipid annotations, as the absence of expected fragment ions may result from analytical limitations rather than the true absence of the corresponding compounds.

The coupling between isolation window configuration and spatial sampling (Figure 2) defines a central methodological trade-off. Increasing isolation window width improves spatial sampling frequency but also increases spectral complexity due to enhanced cofragmentation. Conversely, increasing the number of narrower isolation windows improves mass selectivity but enlarges the spatial distance between repeated acquisitions of a given *m*/*z* range, so small localized tissue regions may not be sampled by matching MS^2^ spectra, and compounds confined to very localized areas can therefore remain undetected. Thus, DIA window design must balance spectral clarity, spatial fidelity, and mass range coverage according to the specific analytical objective.

In the present study, data were acquired using the 2D pixel mode of the SMALDI control software. This mode was chosen because it enables straightforward assignment of spectra to pixel coordinates and thereby facilitates image reconstruction. However, acquisition speed could be substantially increased by switching to 2D line-scan mode, which reduces per-pixel overhead while maintaining sufficient spatial fidelity.^31^ Additional acceleration could be achieved using faster mass spectrometers. Recent developments demonstrate that coupling an AP-SMALDI source with an Orbitrap ASTRAL mass spectrometer can substantially improve acquisition speed and overall efficiency.^32^

A further limitation of the presented approach is the reduction in effective spatial resolution compared with conventional MS^1^-based MSI. Because DIA MS^2^ spectra rather than MS^1^ spectra are acquired at specific sample positions, the resulting MS images display lower spatial detail. Nevertheless, the acquired DIA MS^2^ spectra provide complementary fragmentation information that improves confidence in compound and lipid-group annotations.

## Conclusions

In this study, a DIA-based MALDI MSI workflow was developed that combines spatially resolved MS^1^ imaging with complementary DIA MS^2^ fragmentation information. The approach integrates randomized DIA acquisition, precursor-associated MS^2^ analysis, and spatially informed annotation strategies within an extended Compound Discoverer workflow. Application to *F. hepatica* tissue demonstrated that combining precursor mass information, DIA MS^2^ spectra, and spatial-correlation filtering can improve compound and lipid-group annotations compared with MS^1^-only MSI.

At the same time, the study highlights important methodological trade-offs between spectral complexity, spatial sampling density, and effective spatial resolution that arise from DIA window design. Although the presented workflow does not provide complete DIA spectral deconvolution or unambiguous structural elucidation for all compounds, it demonstrates that DIA fragmentation information can be integrated effectively into MSI workflows to support spatially resolved compound annotation and retrospective data analysis.
